# Evidences of HEV genotype 3 persistence and reactivity in liver parenchyma from experimentally infected cynomolgus monkeys (*Macaca fascicularis*)

**DOI:** 10.1371/journal.pone.0218472

**Published:** 2019-06-18

**Authors:** Diana Chaves Pereira Mejido, Jaqueline Mendes de Oliveira, Ana Maria Coimbra Gaspar, Noemi Rovaris Gardinali, Fernanda de Oliveira Bottino, Lilian Gonçalves de Carvalho, Debora Regina Lopes dos Santos, Yohan Brito Kevorkian, Leandro Layter Xavier, Julio Moran, Marcelo Pelajo-Machado, Renato Sergio Marchevsky, Marcelo Alves Pinto

**Affiliations:** 1 Laboratory of Technological Development in Virology, Oswaldo Cruz Institute, Oswaldo Cruz Foundation, Rio de Janeiro, Brasil; 2 Institute of Science and Technology of Biomodels, Oswaldo Cruz Foundation, Rio de Janeiro, Brasil; 3 Departament of Veterinary Microbiology and Immunology, Federal Rural University of Rio De Janeiro, Rio de Janeiro, Brasil; 4 Laboratory of Morphometry, Institute of Biology, Rio de Janeiro State University (UERJ), Rio de Janeiro, Brasil; 5 Laboratory of Pathology, Oswaldo Cruz Institute, Oswaldo Cruz Foundation, Rio de Janeiro, Brasil; 6 Dr Julio Moran Laboratories, Ebmatigen, Zurich, Switzerland; 7 Laboratory of Control of Neurovirulence, Bio-Manguinhos, Fundação Oswaldo Cruz, Rio de Janeiro, Brasil; CEA, FRANCE

## Abstract

Hepatitis E virus genotype 3 (HEV-3) is an emerging zoonotic pathogen, responsible for sporadic cases of acute hepatitis E worldwide. Primate models have proven to be an essential tool for the study of HEV pathogenesis. Here we describe the outcomes of HEV infection in *Macaca fascicularis* (cynomolgus) inoculated experimentally with genotype 3. Eight adult cynomolgus macaques were inoculated intravenously with HEV-3 viral particles isolated from swine and human samples. Liver, spleen, duodenum, gallbladder and bile were sequential assessed up to the end-point of this study, 67 days post-inoculation (dpi). Our previously published findings showed that biochemical parameters return gradually to baseline levels at 55 dpi, whereas anti-HEV IgM and HEV RNA become undetectable in the serum and feces of all animals, indicating a non-viremic phase of recovery. Nevertheless, at a later stage during convalescence (67 dpi), the presence of HEV-3 RNA and antigen persist in central organs, even after peripheral viral clearance. Our results show that two cynomolgus inoculated with swine HEV-3 (animals I3 and O1) presented persistence of HEV RNA low titers in liver, gallbladder and bile. At this same stage of infection, HEV antigen (HEV Ag) could be detected in all infected animals, predominantly in non-reactive Kupffer cells (CD68+iNOS-) and sinusoidal lining cells. Simultaneously, CD4+, CD3+CD4+, and CD3+CD8+ immune cells were identified in hepatic sinusoids and small inflammatory clusters of lobular mononuclear cells, at the end-point of this study. Inability of HEV clearance in humans can result in chronic hepatitis, liver cirrhosis, with subsequent liver failure requiring transplantation. The results of our study support the persistence of HEV-3 during convalescence at 67 dpi, with active immune response in NHP. We alert to the inherent risk of viral transmission through liver transplantation, even in the absence of clinical and biochemical signs of acute infection. Thus, besides checking conventional serological markers of HEV infection, we strongly recommend HEV-3 RNA and antigen detection in liver explants as public health measure to prevent donor-recipient transmission and spread of hepatitis E.

## Introduction

In Brazil and other Latin America countries, hepatitis E is considered a viral emerging zoonotic disease, with HEV-3 genome strain circulating mainly among pig herds [1] and immunocompromised patients [2]. Our group reported the first autochthonous case in Brazil and until the moment, HEV infection is rarely detected in sporadic clinical cases [3]. Acute hepatitis E is clinically indistinguishable from hepatitis A, another enterically transmitted viral hepatitis, being both characterized as self-limited inflammatory liver diseases [4]. Regarding pathogenesis of hepatitis E, it is well known that potent innate and adaptative immune responses, driven specially by CD4 and CD8 T-cells to ORF2 protein (capsid), are correlates of protection against HEV infection [5]. Immunocompromised subjects displays a weaker specific T-cell response, which is associated with chronic form of HEV infection [6]. HEV replication in liver transplanted patients under immunosuppressive therapy can also lead to liver failure, cirrhosis and chronic hepatitis, mimicking acute graft rejection [7].

HEV infection in nonhuman primate (NHP) models, mainly in cynomolgus monkey (*Macaca fascicularis*), reproduce several features of human hepatitis E, including biochemical changes in serum and liver inflammation, observed in both subclinical [8][9] and chronic cases [10]. Domestic pigs are intriguing but still underexplored animal model [11]. Low viral titers of HEV persists in liver parenchyma of naturally infected swine, even after conventional serological markers became undetectable [12]. Thus, ingestion of contaminated food by viscera [13], as well as liver xenotransplantation [14] represent a potential viral source of acute or chronic hepatitis E, respectively [15].

In this study, we investigated the persistence of HEV in liver parenchyma and extra-hepatic sites during recovery, after spontaneous remission of immunocompetent monkeys infected with HEV-3 strain. We also evaluated histological markers of inflammation and cellular immune response involved in intrahepatic compartment during convalescence.

## Material and methods

### Animals and ethics statement

Ten clinically healthy young adult cynomolgus monkeys, each weighing 1.5–6.0 kg, aged 2 to 19 years-old, provided from the breeding colony of the Primatology Service of the Institute of Science and Technology on Biomodels (ICTB), Fiocruz, Rio de Janeiro, Brazil. The study protocol was approved (L-0033/07) by the Institutional Animal Care and Use Committee (CEUA-Fiocruz), conducted in strict accordance with recommendations from the Guide for the Care and Use of Laboratory Animals of the Brazilian Society of Science in Laboratory Animals (SBCAL) and the National Animal Experimentation Control Board (CONCEA, Brazilian Ministry of Science and Technology). The animals selected for the study were free of simian immunodeficiency virus (SIV), simian type D retrovirus (SRV/D) and negative for anti-HEV IgG. Absence of histological liver changes was confirmed by pre inoculation analysis of liver biopsies. During the study and quarantine periods, monkeys were housed in indoors research facilities (Animal Biosafety Level 2) at the Coordination of Research in Animal Experimentation (ICTB/Fiocruz), and kept individually in stainless steel squeeze-back cages (0.77 m height x 0.60 m width x 0.68 m depth) in climate-controlled rooms (temperature of 22 ± 1°C and humidity 55 ± 5%) with a 12 h light/dark cycle. Animals were fed with a commercial primate diet supplemented with fresh fruits and vegetables. Water was provided *ad libitum*. In order to minimize the stress of the animals throughout the study period, an environmental enrichment program was implemented, consisting of a series of measures that modify physical and social aspects, improving the quality of housing and life of animals, such as: (i) stainless steel mirror—made of polished stainless steel attached to the front cage grid, allowing the animal to move it to explore the environment; (ii) foraging tray—made of stainless steel containing a plastic carpet with recesses attached to the cage, allowing the animal to handle some items (i.e., cereal bar fragments, raisins, sunflower seeds) arranged on the tray; (iii) PVC of biting—made with PVC pipe with perforations along its length and threaded caps at the ends, which allow the placement of items (i.e., cereal bar pieces, raisins, rice grains); (iv) electronic equipment offering classical music in CD’s; (v) microwave popcorn—popcorn offer for at least one day of the week; (vi) medicinal herbs—offering various medicinal herbs in at least one week day (mint, lemon balm, chamomile, and calendula).

For the collection blood and pre-inoculation liver biopsies, the animals were anaesthetized with ketamine hydrochloride at 20 mg/kg (Vetanarcol, König, Argentina), xylazine hydrochloride at 0.1 mg/kg (Syntec Brazil, São Paulo, Brazil) and midazolalam hydrochloride at 1.0mg/kg (Roche, Farmacêutica do Brasil, SP, Brazil). All animals were euthanized under deep barbiturate anaesthesia with sodium thiopental 2.5% at 40 mg/kg (Thiopentax, Cristalia, São Paulo, Brazil), which was delivered intravenously. Subsequently, cardiac punctures were performed, and the animals were euthanized by exsanguination at 67 dpi.

### Experimental design

Eight adult *Macaca fascicularis* were inoculated intravenously with swine HEV genotype 3 strain (Dutch and Brazilian cases—10^5−6^ copies/mL) or human (Argentine and Brazilian 10^5^copies/mL), whereas another two control animals received phosphate-buffered saline (PBS, 10%) solution (pH 7.3). The Brazilian swine inoculum was HEV genotype 3 strain (GenBank EF591853.1) isolated from fecal suspension obtained from a naturally infected pig breeding in a commercial farm in Rio de Janeiro [1]. The Dutch swine HEV genotype 3 strain (D-swine) (GenBank DQ996399) was kindly supplied by the Central Veterinary Institute of Wageningen University and Research Centre, the Netherlands [16]. The Brazilian human HEV genotype 3b strain (Br-human) (GenBank GQ421465) was isolated from 1ml serum sample obtained from a 30-year-old patient with acute hepatitis E [3]. The Argentinean human HEV sample (Ar-human) was kindly provided by Dr. Carlos Malbran Institute, Buenos Aires, prepared from a pool of 1ml of serum and faeces from a 3-month-old patient with fulminant acute hepatic failure. This study was approved by the institutional review boards (CEP-Fiocruz No. 22/03).

**Fig 1:**
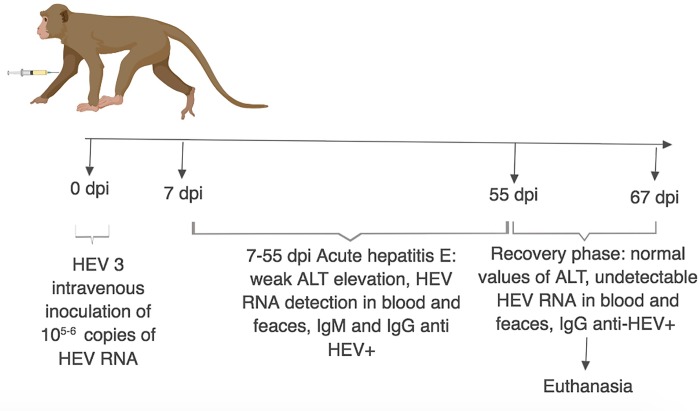
Study experimental design. Liver samples of HEV infected monkey assessed after hepatitis E convalescence and cure (67 dpi).

Monkeys were followed up during 67day post-inoculation (dpi) by veterinary clinical care (daily), with periodic assessment of biochemical and virological parameters (Data showed in our previously published study) [8]. Pre- and post-inoculation sera were tested for macaque anti-HEV IgG and IgM using a modified protocol (Dr. Julio Moran Laboratories, Zurich, Switzerland) from commercially available Diacheck anti-human HEV antibody assay, using adapted goat anti-macaque immunoglobulin conjugate (Fitzgerald Industries International Inc., USA). Pre-inoculation liver biopsies were performed in all animals in order to confirm absence of liver injury as previously described [8] [17]. All animals reached baseline parameters at 55 dpi, similarly to those obtained at pre-inoculation step (normal parameters). Euthanasia, under deep anesthesia and analgesia, and necropsy were performed at 67 dpi, as previously described [18]. In this study we accessed samples at 67 dpi, collected after euthanasia and after virological and biochemical cure (recovery) considered at 55 dpi according to our previous data in acute phase of infection [8] (**Fig 1**).

#### Liver histopathology

A large amount of tissue samples was collected during necropsy and extensively assessed, including serum, urine, stool, bile, and tissue fragments of liver, spleen, duodenum and gallbladder. Samples were frozen and stored at -70°C until analysis. A portion of each sample was stored in 10% buffered formalin (pH 7.0), before processing and embedded in paraffin according to standard methods. Paraffin blocks were sectioned at 4μm and stained with haematoxylin-eosin. Slides were examined under brightfield microscopy. Inflammatory lesions were quantified using a scale from 0 to 4 based on the number of focal mononuclear cell infiltrates per 10 hepatic lobules. The following scale was set up: 0 = no inflammation, 1 = 1 to 2 focal infiltrates (poor), 2 = 3 to 5 focal infiltrates (mild), 3 = 6 to 10 focal infiltrates (moderate), and 4 = over 10 focal infiltrates (severe) as described previously [19]. All technical histological procedures were performed in the Laboratory of Neurovirulence Control (LANEU/Bio-Manguinhos, Fiocruz).

Animals were grouped in three experimental conditions according to the inoculum, referred as: a) Swine HEV-3: inoculum obtained from Brazilian pigs infected naturally (I3, Q11 and X15) or from Dutch pigs infected experimentally (O1, G3 and F3); b) Human HEV-3: inoculum obtained from a Brazilian patient with acute hepatitis E (J3) or from an Argentine human patient with fulminant hepatitis (R7), and c) Control: animals (I2 and Q12) inoculated with phosphate-buffered saline solution (PBS, 10%).

#### HEV RNA detection and quantification

HEV viral load was assessed by Taq Man real time qPCR in liver, spleen, duodenum, gallbladder, bile, urine, serum and fecal samples obtained 67dpi, during the convalescence period. Total RNA was extracted from 140 μL of serum, urine, fecal suspensions (10% w/v in PBS) and bile (1:10 in RNAse free water) using the QIAamp Viral RNA Kit (Qiagen, USA); and from 30 mg of tissue fragments using the RNeasy kit, Qiagen Mini Kit (Qiagen, USA). Positive controls included fecal suspensions of naturally HEV-infected pigs and fragments of liver biopsy obtained from experimentally infected pigs, donated by Dr. Van Der Poel from the Veterinary Department, Institute of Wageningen University and Research Centre, Wageningen, Netherlands. Reverse transcription (RT) and real-time PCR (q-PCR) reactions were carried out as previously described [8]. Reactions were performed in duplicate for each sample, using 25 μL of final volume, corresponding to 5 μL of cDNA, 12.5 μL of TaqMan Universal PCR Master Mix (Life Science Technologies, USA), 6.5 μL of RNase free water and primers and probe as described previously [20]. A standard curve generated with plasmid cloned from a Brazilian swine strain characterized previously as HEV-3 positive sample was used to provide quantification parameters (serial dilutions from 101–10^7^) [8]. For nested PCR reaction, oligonucleotide primers targeting HEV ORF2 region were used according to the protocol previously described [21].

To identify HEV replication sites, tissue samples with detectable positive-strand HEV RNA were also tested for detection of HEV RNA negative-sense by ORF2 nested RT-PCR protocol. For this purpose, the extracted RNA was reverse transcribed with SuperScript III reverse transcription (Invitrogen Life Technology, CA, USA), in the presence of the external forward primer from ORF2 protocol region [21]. Then, PCR and nested PCR were carried out to amplify partial fragment of ORF2.

#### HEV antigen and immune cells detection

All frozen liver sections (4μm), obtained at 67 dpi, were examined by indirect immunofluorescence (IFI). To assess HEV Ag, a rabbit anti-HEV polyclonal antibody at a 1:150 dilution (Fitzgerald Industries International Inc., USA) was used as primary antibody (1 mg/mL), followed by a FITC-labelled goat anti-rabbit IgG antibody (2 mg/mL) at a 1:750 dilution (Sigma-Aldrich, USA) as secondary antibody. Liver sections were counterstained with Evans Blue (1:20.000), mounted with ProLong Gold or SlowFade Gold reagents in glycerol with DAPI (Life Technologies, USA), and covered with a coverslip. To assess cell phenotypes, frozen liver sections (4μm) were assayed with the following commercial antibodies as primary antibodies: polyclonal rabbit anti-CD68 (Abcam, UK), monoclonal mouse anti-iNOS (LifeSpan Biosciences, USA), monoclonal rat anti-CD3 (Serotec Info, Brazil), monoclonal mouse anti-CD4 (BD Pharmigen, USA), and monoclonal mouse anti-CD8 (BD Pharmigen, USA). Secondary antibodies to distinguish different phenotypes were employed: Alexa Fluor 647-labeled polyclonal goat anti-rabbit IgG, and Alexa Fluor 488-labeled polyclonal goat anti-mouse IgG (Abcam, UK). The images of labelled tissues were obtained by confocal microscopy (LSM Zeiss 510 META, Carl Zeiss, Germany) in the Laboratório de Imagem, Universidade Estadual do Rio de Janeiro (UERJ), Rio de Janeiro, Brasil.

#### Statistical analyses

Comparison among groups was carried out using average and standard deviation (x±sd). Besides control group parameters, pre-inoculation values were also used as baseline values since all monkeys were healthy and not infected by hepatitis E virus in the past. Average of each cell phenotype was compared using the non-parametric Kruskal-Wallis test, with Dunn’s Multiple Comparison Test. The level of significance adopted was of p ≤ 0.05 with the aid of Prism 5 program (GraphPad software, USA).

## Results

### Histopathological features of liver from cynomolgus monkeys infected with HEV

Histological analyses of liver biopsies at pre-inoculation period were considered normal in all animals. Animals inoculated with Human HEV-3 (R7 and J3) showed liver focal inflammatory reaction (final scores 0 to 1) at 67 dpi. Animal R7 showed microsteatosis (+1), steatosis (+1), inflammatory focus (+1), portal inflammation (+1) and J3 showed also microsteatosis (+1) and inflammatory focus (+1), besides the presence of Ito cells (+1). Similarly, monkeys inoculated with Swine HEV-3 (O1, G3, F3, I3, Q11 and X15), exhibited changes compatible to histological HEV-related hepatitis at 67 dpi (final scores ranging 1 to 3). Control group (I2 and Q12) showed minor liver inflammatory findings at 67 dpi (final scores 0) (**S1 Table)**. The experimental groups; histopathological final scores of hepatic lesions at 67dpi; previous findings of HEV RNA detection in serum and faecal samples [8] and the detection of HEV RNA and antigen during convalescence period in liver (67 dpi) are summarized in **Table 1**.

**Table 1:** Distribution of cynomolgus HEV infected and control groups; summarized findings of hepatic lesions and HEV RNA / antigen detection in liver lesions at 67 dpi.

Groups	NHP (ID)*	Age (years)	Hepatic lesions 67 dpi* (scores)**	HEV Ag	HEV RNA (dpi)***		
				Liver	Serum	Feces	Liver
*Swine HEV-3*	I3	15	3	+	39–53	21–53	+
	Q11	18	1	+	14	7–21	-
	X15	2	2	+	—-	14–21	-
	O1	11	1	+	14	5–18	+
	G3	17	1	+	7–14	7–18	-
	F3	19	1	+	—-	—-	-
*Human HEV-3*	R7	7	0	+	—-	14–27	-
	J3	14	1	+	—-	—-	-
Control	I2	16	0	**-**	—-	—-	-
	Q12	18	0	**-**	—-	—-	-

* ID, identification; dpi, days post inoculation

** Liver sections were prepared from samples collected during necropsy; liver injury score: 0 –without inflammation, 1–1 to 2 lymph histiocytic inflammatory infiltrates/10 liver lobules, 2–3 to 5 inflammatory infiltrates/10 lobules, 3–6 to 10 inflammatory infiltrates/10 lobules, 4- >10 inflammatory infiltrates/10 lobules [19].

*** Complementary data of HEV RNA detection in serum and feces (performed in our previously published study) [8].

**Fig 2:**
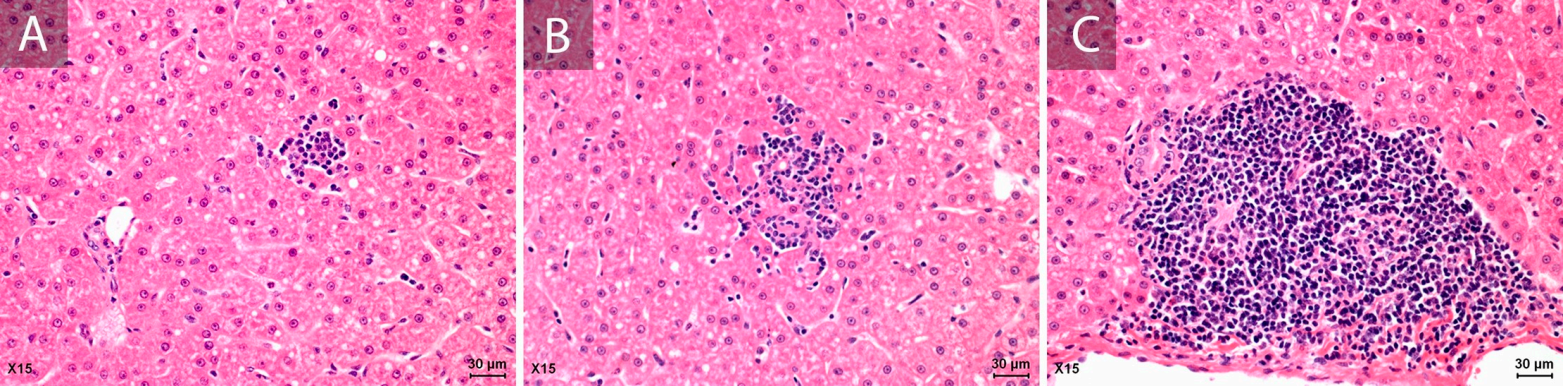
Representative histological liver features of cynomolgus monkeys (X15) infected with Swine HEV-3 at 67 dpi. Hematoxylin-eosin (H&E) (A, B and C). (A) Focal collection of mononuclear cells and mild microsteatosis; (B) Cluster of lobular lymphocytes and macrophages and (C) Mild mononuclear cells infiltration of portal tract.

The best representative inflammatory liver injury was demonstrated in X15 liver samples and showed at **Fig 2A–2C**. Slight to mild fatty changes and mild diffuse hepatocyte swelling were detected in all animals, even with or without inflammatory response.

### HEV RNA load distribution through different tissues in absence of biochemical and virological markers of liver damage

Viral RNA was performed in samples of serum, bile, faeces, urine, liver, spleen, duodenum, and gallbladder by real time qRT-PCR and by nested PCR at the end-point of this study (67 dpi). Positive samples were subsequently tested by nested PCR for sequencing (**S1 Fig**). HEV RNA were observed in gallbladder (O1 and I3) and bile (I3) samples, both detected in lower titers, between 10^1^ and 10^3^ copies/mL, at 67 dpi, in two cynomolgus monkeys infected with Swine HEV-3 as shown in **Table 2**. We could detect HEV RNA in the liver of animal O1 only by Nested PCR (**S1 Fig**). HEV RNA was undetectable in both control and Human HEV-3 strain groups at 67 dpi.

**Table 2:** Genomic HEV RNA detection in tissue samples at 67 days post-inoculation.

Animal (ID)*	Inoculum description	qPCR detection			Nested PCR
		Positive samples	Viral load (RNA/gr or mL)	Ct*	
I3	Swine HEV3	Gallbladder	6 x 10^1^	40.7	+
		Bile	2.1 x 10^2^	38.5	+
O1		Gallbladder	5.3 x 10^3^	34.3	+
		Liver	-	-	+

* ID, identification; Ct, Cycle threshold

All positive samples were assayed to minus intermediate-RNA HEV, with undetectable results. Sequencing of amplified products confirmed that RNA found in samples corresponded to hepatitis E virus (GenBank GQ421465). Results were summarized at **Table 1**.

### HEV antigen (HEV Ag) detection and inflammatory cell reactivity in liver parenchyma

**Fig 3:**
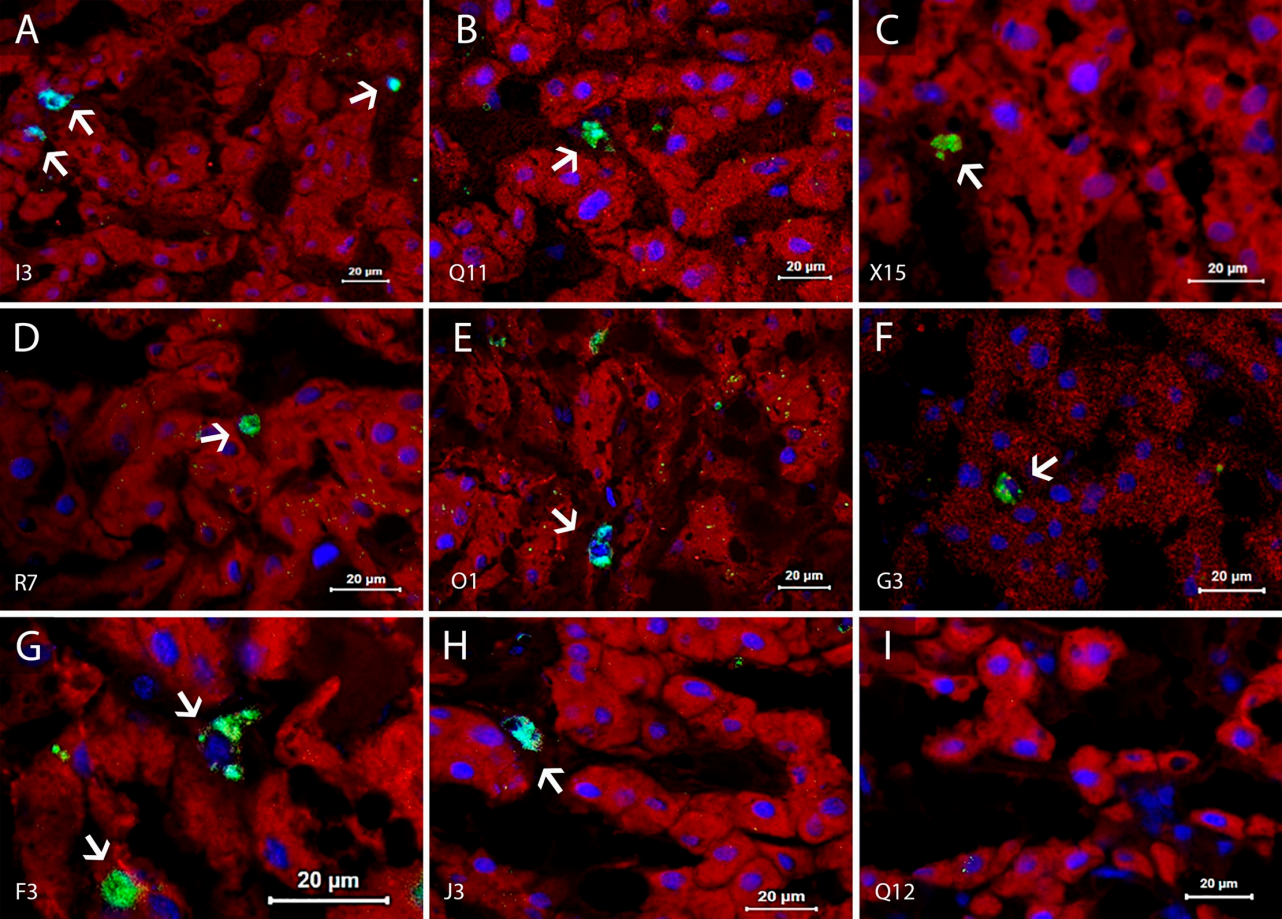
HEV antigen detection in liver sections from HEV infected monkeys at 67 days post-inoculation. Presence of HEV Ag (white arrows) was detected by indirect imunofluorescence (IFI). (A-D and F-H) HEV Ag labeled cells in hepatic sinusoid (I3, Q11, X15, R7, G3, F3, and J3); (E) cytoplasmic HEV Ag staining evidenced in sinusoidal circulating cell (O1); (I) Absence of HEV+ cells in the liver of a control animal (Q12). Cynomolgus’s identification is displayed on the bottom left-hand side. Anti-HEV Ag/FITC (green and cyan); Nuclei stained with DAPI (blue) and liver parenchyma counterstained with Evans blue (red).

All infected animals exhibited positivity for HEV Ag in the liver at 67 dpi. Presence of HEV Ag was evidenced in cytoplasm of hepatocytes, sinusoidal lining cells and circulating mononuclear cells **(Fig 3A–3H)**. Control animals were negative as expected and are represented in **Fig 3I**.

**Fig 4:**
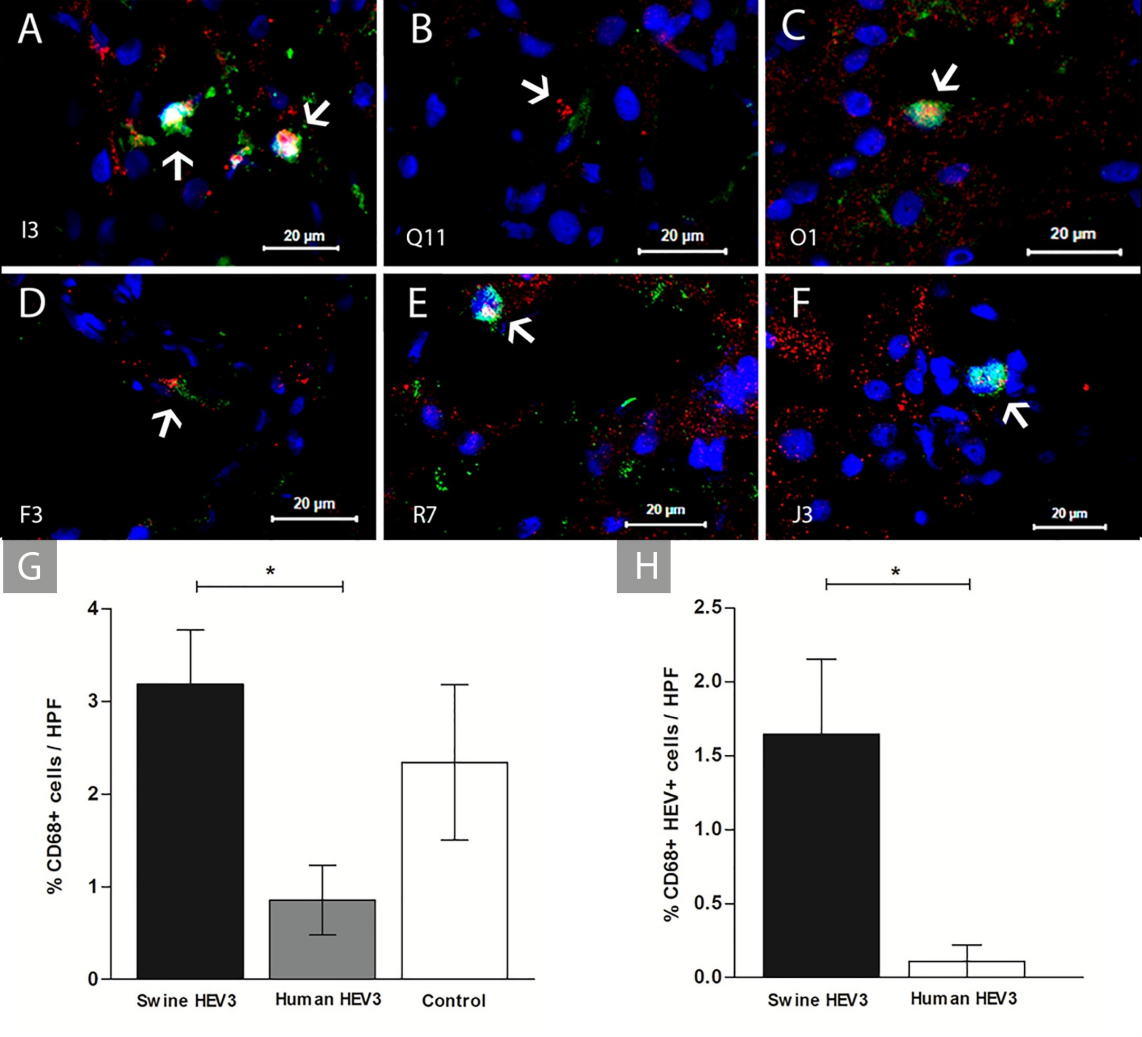
Double staining for HEV antigen of liver sections and CD68+ cells of infected cynomolgus monkey. Infected Kupffer cells (CD68+HEV+) (white arrow) in hepatic sinusoids detected by IFI. **(A-D)** Labeled CD68+HEV+ sinusoidal cells of animals I3, Q11, O1, and F3 (Swine HEV-3); **(E-F)** Labeled CD68+HEV+ sinusoidal cells of animals R7 and J3 (Human HEV-3). Cynomolgus’s identification is displayed on the bottom left-hand side. Anti-CD68/Alexa fluor 647 (red), Anti-HEV Ag/FITC (green), co-staining CD68+HEV+ (yellow—regions of overlap) and Nuclei stained with DAPI (blue); **(G)** Frequency of Kupffer cells (CD68+) in the hepatic sinusoids of experimental groups (Swine HEV-3 [n = 6], Human HEV-3 [n = 2] and Control [n = 2]); **(H)** Frequency of Kupffer cells with HEV (CD68+HEV+) from hepatic sinusoids experimental groups (Swine HEV-3 [n = 6] and Human HEV-3 [n = 2]). Bars represent the means with standard error. Selection marked with * characterizes differences between groups analyzed with non-parametric Kruskal-Wallis test and Dunn’s Multiple Comparison (0.01 ≤ p <0.05). HPF, high-power field.

The quantification of cell types staining is shown at **S2 Table** (raw data). Intra-hepatic inflammatory cell reactivity was confirmed by the presence of Kupffer cells and in scattered in the mononuclear cells throughout lobular or portal tracts. Double labelling revealed HEV Ag associated with Kupffer cells (CD68+ HEV+) **(Fig 4A–4F)**, Kupffer cells (CD68+) counts showed absence of differences between infected and control groups (p = 0.0583) **(Fig 4G)**. With respect to the source of HEV (swine and human), a reduction in the frequency of CD68+ cells occurred in animals inoculated with Human HEV-3 (0.01 ≤ p <0.05) **(Fig 4G)**. CD68+HEV+ cells in infected animals were predominant in the group inoculated with Swine HEV-3 (p = 0.0293) **(Fig 4H)**. Ratio of CD68+HEV+ over the total of CD68+ cells was shown to be four times greater in animals inoculated with Swine HEV-3 than those that received Human HEV-3 as inoculum **(Fig 4H)**.

**Fig 5:**
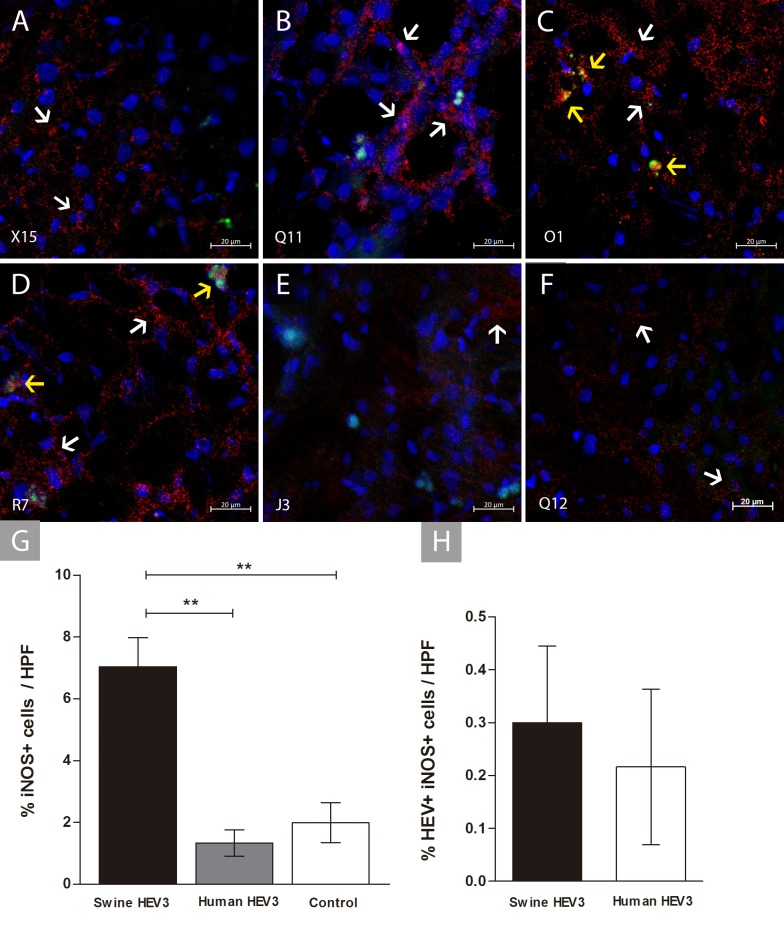
Double staining of liver sections for HEV antigen and iNOS of infected cynomolgus monkey. Detection of positive staining for iNOS (white arrows) and HEV infected cells producing nitric oxide (HEV+iNOS+) in the hepatic parenchyma (yellow arrows). **(A-C)** Nitric oxide (NO) produced by hepatocytes of animals X15, Q11 and O1 (Swine HEV-3); **(D-E)** NO produced by liver cells of animals R7 and J3 (Human HEV-3); **(F)** Scarce cells iNOS+ from the hepatic parenchyma of Q12 (Control). Cynomolgus’s identification number is observed on the bottom left-hand side. Antibody anti-iNOS/Alexa fluor 647 (red), anti-HEV/FITC (green), co-staining HEV+iNOS+ (yellow—regions of overlap) and DAPI stained nuclei (blue); **(G)** Frequency of iNOS-producing cells in the hepatic parenchyma from the experimental groups (Swine HEV-3 [n = 6], Human HEV-3 [n = 2] and Control [n = 2]). Bars represent means with standard error. Selection marked with * characterizes differences between groups (0.01 ≤ p <0.05); **(H)** Frequency of HEV+iNOS+ cells in the hepatocytes from the experimental groups (Swine HEV-3 [n = 6] and Human HEV-3 [n = 2]). Bars represent means with standard error and no significant difference was observed between groups (p = 0.9867). Results analyzed with non-parametric Kruskal-Wallis and Dunn’s Multiple Comparison test. HPF, high-power field.

For assessment of HEV-induced inflammatory activation in liver parenchyma, inducible nitric oxide enzyme (iNOS) production was investigated. Parenchymal and sinusoidal cells producing iNOS (iNOS+) were recorded in all groups, predominantly in infected animals (P <0.0001) (**Fig 5A–5F**). Double staining of infected cells producing iNOS (HEV+iNOS+) are represented at **Fig 5C and 5D.** Regarding the source of inoculum, the highest iNOS+ cells frequency occurred in the group inoculated with Swine HEV-3 (0.001≤ p <0.01) **(Fig 5G)**. Absence of double-labelled CD68+iNOS+ cells in all tested liver section was registered. Presence of HEV+iNOS+ double labelled cells showed no significant differences when comparing the source of inoculum in infected groups (p = 0.9867) **(Fig 5H)**.

**Fig 6:**
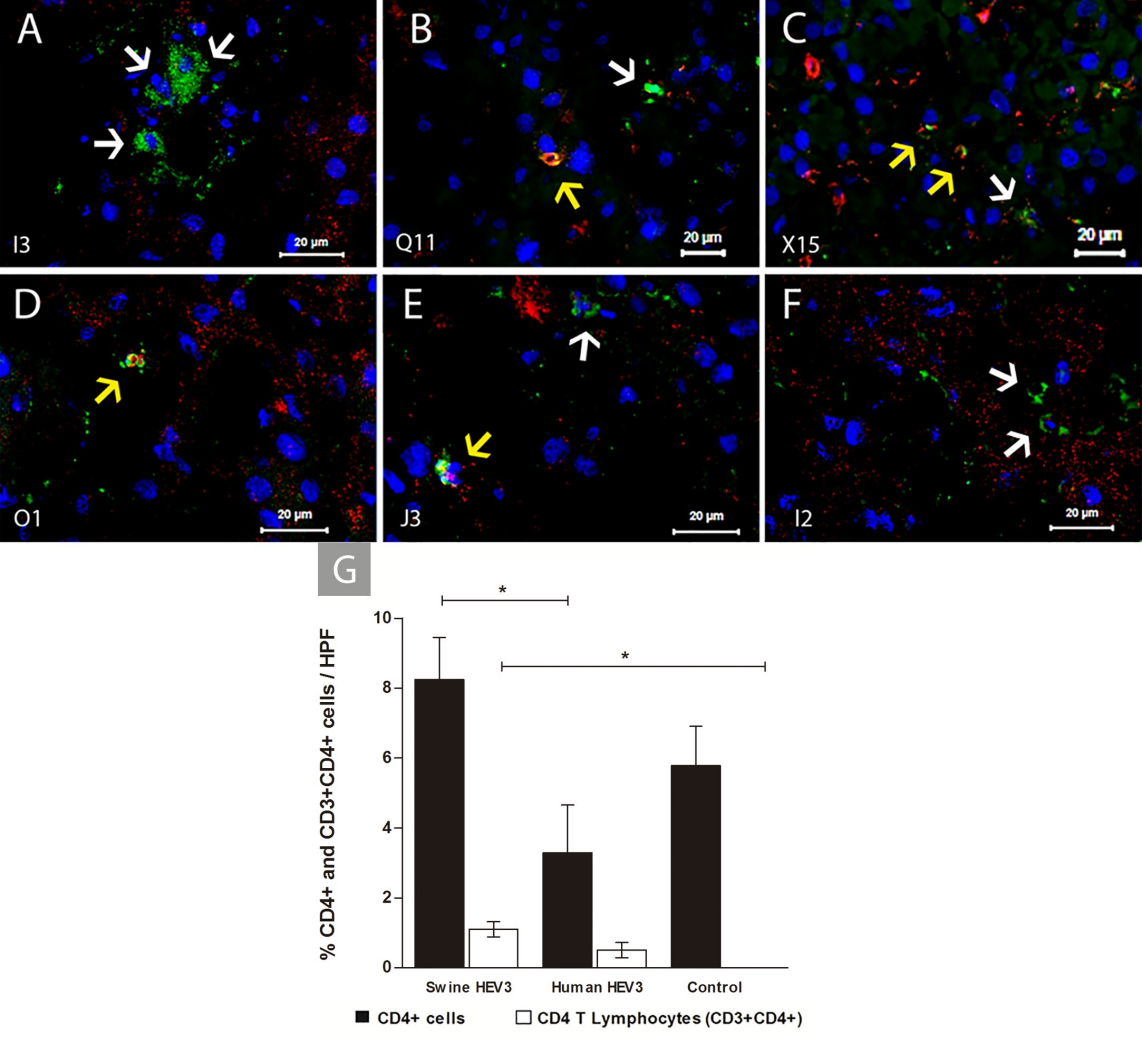
Immunofluorescence detection of CD4+ lymphocytes at 67 dpi in liver sections of HEV infected cynomolgus monkey. Detection of CD4+ cells (white arrow) and lymphocytes T CD4 (CD3+CD4+) (yellow arrow) by IFI. **(A)** CD4+ cells (I3—Swine HEV-3); **(B-D)** CD4+ cells and T CD4 lymphocytes (Q11, X15, and O1—Swine HEV-3); **(E)** CD4+ cells and CD4 T lymphocytes in liver parenchyma (J3—Human HEV-3); **(F)** CD4+ cells (I2—Control). Monkeys’s identification number is shown on the bottom left-hand side. Anti-CD3/Alexa fluor 647 (red), Anti-CD4/FITC (green), co-staining CD3+CD4+ (yellow—regions of overlap) and Nuclei stained with DAPI (blue). **(G)** Frequency of CD4+ cells and CD4 T lymphocytes (CD3+CD4+) in hepatic parenchyma from the experimental groups (Swine HEV-3 [n = 6], Human HEV-3 [n = 2] and Control [n = 2]). Bars represent mean values of standard error values. Selection marked with * characterizes a significant difference between groups analyzed with non-parametric Kruskal-Wallis test and Dunn’s Multiple Comparison (0.01 ≤ p <0.05). HPF, high-power field.

Lymphocytic infiltrates were identified and quantified in liver parenchyma. We found increased frequency of CD4+ cells in infected animals (p = 0.0170), greater in animals inoculated with Swine HEV-3 than those receiving Human HEV-3 (0.01 ≤ p <0.05). Infected animals displayed magnification of CD4 T lymphocytes (CD3+CD4+) frequency (p = 0.0085). Significantly, CD4+T cells were greater in cynomolgus inoculated with Swine HEV-3 when compared to control group (0.01 ≤ p < 0.05) **(Fig 6A–6G)**.

**Fig 7:**
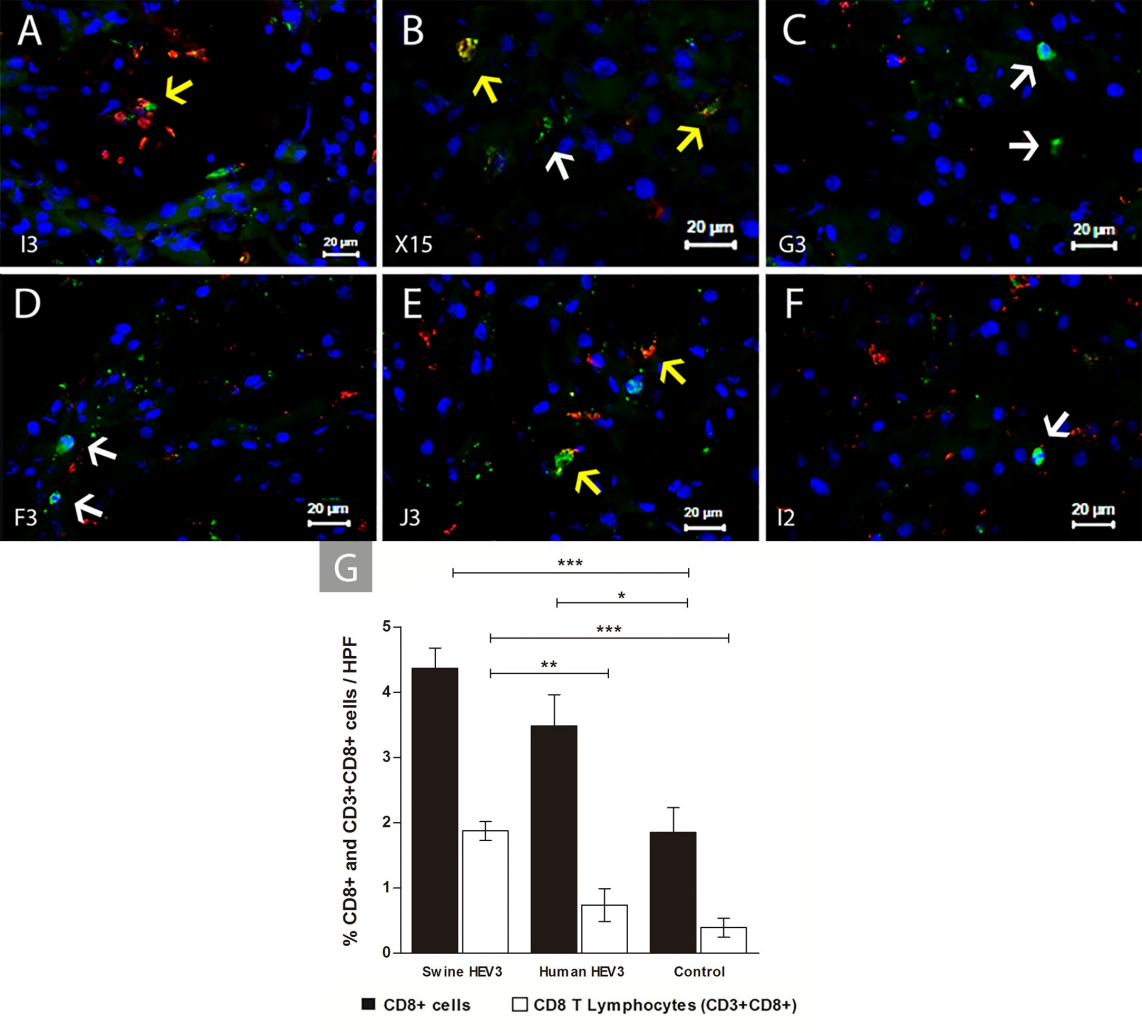
Immunofluorescence detection of CD3+ and CD8+ lymphocytes at 67 dpi in liver sections of HEV infected cynomolgus monkey. Detection of CD8+ cells (white arrow) and CD8+ T lymphocytes (CD3+CD8+) (yellow arrow) by IFI. **(A)** CD8 T lymphocytes (I3—Swine HEV-3); **(B-D)** CD8+ cells and T CD8 lymphocytes (X15, G3 e F3—Swine HEV-3); **(E)** T CD4 lymphocytes (J3—Human HEV-3); **(F)** CD8+ cell (I2—Control). The monkeys’s identification is shown on the bottom left- hand side. Anti-CD3/Alexa fluor 647 (red), Anti-CD8/FITC (green), co-staining CD3+CD8+ (yellow—regions of overlap) and Nuclei stained with DAPI (blue) and counterstained with Evans blue (red). **(G)** Frequency of CD8+ cells and CD8 (CD3+CD8+) T lymphocytes in hepatic parenchyma from experimental groups (Swine HEV-3 [n = 6], Human HEV-3 [n = 2] and Control [n = 2]). Bars represent the means with the standard error. Selections with * (0.01 ≤ p <0.05), ** (0.001≤ p <0.01) and *** (p <0.001) represent significant differences between the groups. Results analyzed with non-parametric Kruskal-Wallis and Dunn’s Multiple Comparison test. HPF, high-power field.

Likewise, a higher frequency of CD8 positive cells (CD8+) was observed in infected groups (p = 0.0004), with significant increase in CD8+ cells recorded in monkeys inoculated with swine virus compared to the control group (p <0.001). The same behavior was observed when this cell population frequency in animals inoculated with Human HEV-3 was analyzed, being significantly higher than in control animals (0.01 ≤ p <0.05). CD8 T lymphocytes (CD3+CD8+) frequency was also greater in infected animals (p <0.0001), predominantly in the group inoculated with Swine HEV-3 compared to the group inoculated with HEV of human origin (p = 0.001 to 0.01) and with control group (p <0.001) **(Fig 7A–7G)**.

**Fig 8:**
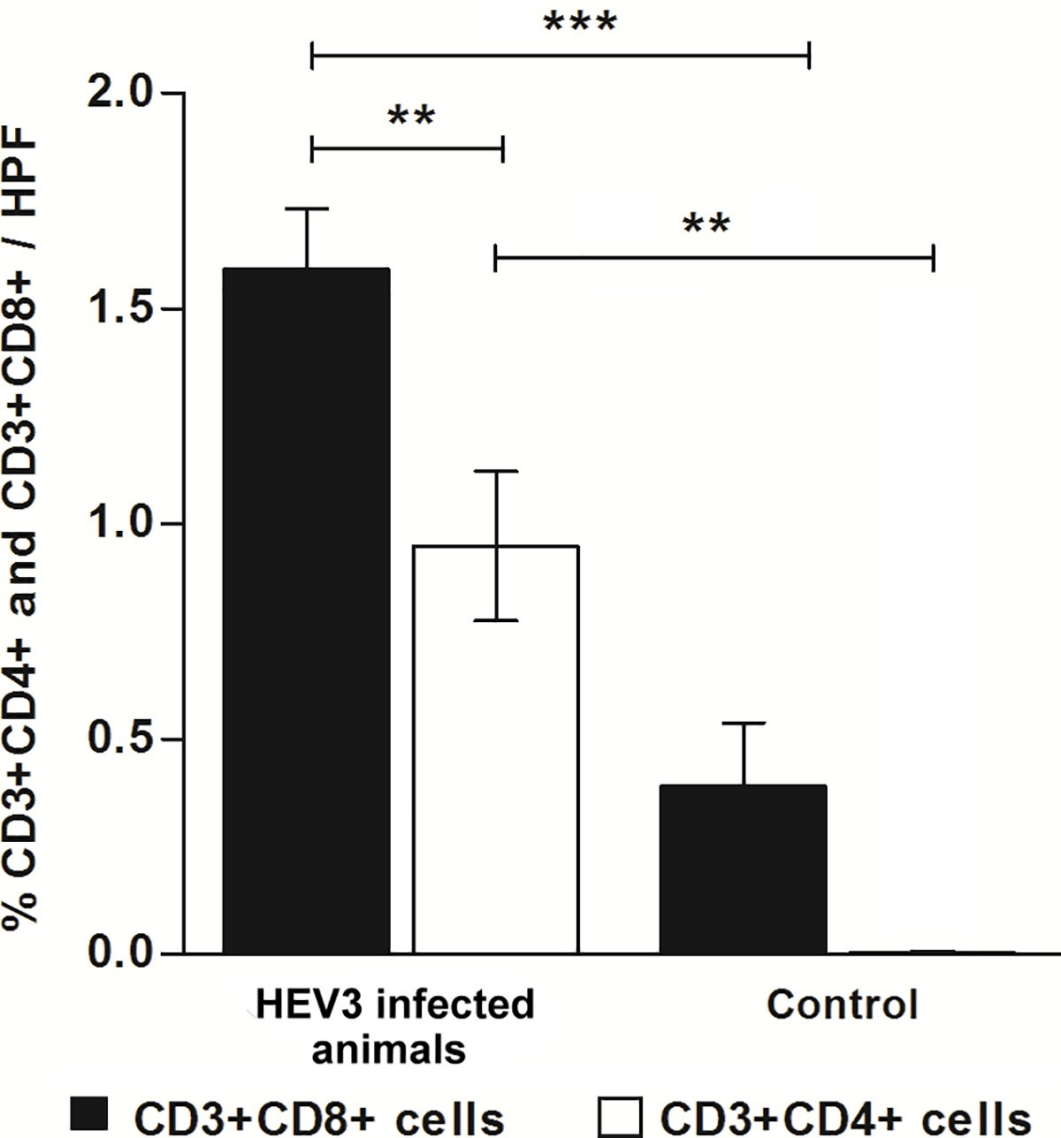
Frequency of CD8 (CD3+CD8+) and CD4 (CD3+CD4+) T lymphocytes in the liver parenchyma of experimentally infected animals and controls. Bars represent the standard error. Selections with ** (0.001≤ p <0.01) and *** (p <0.001) represent significant differences between groups. Results analyzed with non-parametric Kruskal-Wallis and Dunn’s Multiple Comparison test. HPF, high-power field.

Lymphocytic population frequencies were also evaluated considering all infected animals regardless of the inoculum source. Higher frequency of CD4 and CD8 T cell populations in infected animals compared to uninfected animals was noted (0.001≤ p <0.01 / p <0.001). CD8 T population was greater than CD4 T among infected animals (0.001≤ p <0.01). Lymphocytic involvement during the late phase of infection was also proven by CD4T/CD8T ratios (CD3+CD4+/CD3+CD8+), greater in infected animals with both swine (0.59) and Human HEV-3 (0.69) than in control group (0.012) **(Fig 8)**.

## Discussion

Research involving nonhuman primates (NHPs) has proven to be an essential tool for the study of HEV replication and pathogenesis. Here, we described HEV-3 infection in *Macaca fascicularis* (cynomolgus) which elicited virological parameters relevant to evaluating human HEV infection, by using different human and swine sources for experimental infection. Results of our study provide advances in the knowledge of progression of HEV-3 infection.

In our previous study, we observed the absence of HEV RNA in faeces and sera samples, normal levels of ALT, and undetectable anti-HEV IgM in all inoculated cynomolgus monkeys (I3 was the last animal to clear the HEV at 53 dpi), which could support the concept of cure [8]. Here, we observe the persistent detection of lower titles of HEV RNA 67 dpi, in the gallbladder and bile of monkey I3 (inoculated with 10^5^ copies of HEV particles isolated from fecal suspension of pig naturally infected from commercial farms from Rio de Janeiro, Brazil), and gallbladder and liver of animal O1 (inoculated with 10^5−6^ copies of HEV RNA obtained from pigs experimentally infected from Wageningen University, The Netherland). Viral load detected were low in both animals, ranging from 10^1^–10^3^ copies of RNA/ml or g (bile and gallbladder), but still indicates the permanence of virus in the biliary tract, which support the inefficacy of HEV clearance in this advanced phase of infection. Our findings show for the first-time viral RNA persistence for such a prolonged period of infection, during convalescence. The persistence of viral load in the biliary tract could also present a risk of HEV transmission through the ingestion of liver and viscera of poorly processed pig [21].

The minus strain RNA was undetectable in liver parenchyma at 67 dpi, indicating reduced HEV replicative activity, which is consistent with the stage of recovery. However, the presence of HEV Ag was confirmed in liver parenchyma of all inoculated macaques at 67 dpi, during this phase of infection. In our opinion, HEV Ag persistency, predominantly detected in sinusoidal endothelial lining cells, contributed to maintain inflammatory response in liver parenchyma, which together with the presence of hepatocellular tumefaction are compatible with mild viral hepatitis. Previous reports showed persistence of serum and fecal viral shedding in HEV-infected in pigs [22][23] and NHP submitted to immunosuppressive therapy [24].

Concerning HEV-induced inflammatory activity, all infected animals exhibited higher immune cellular reactivity and the increase of iNOS expression. HEV Ag detection and the observed cellular reactivity might contribute to worsening of hepatic injury as described by other authors [25], and could be related with progression of chronic viral hepatitis to cirrhosis, as previously demonstrated in patients with hepatitis B and C [26].

We note that monkeys inoculated with swine HEV-3 showed higher reactivity of CD68+ cells of liver parenchyma, with a clear co-localization of CD68+HEV+ cells, in comparison to those inoculated with human strain. Moreover, the ratio CD68+HEV+/CD68+ cells were four times greater in animals inoculated with swine HEV-3 than in those that received human HEV as inoculum. Also, animals inoculated with swine HEV-3 exhibited the highest frequency of iNOS+ cells, another critical inflammatory marker. Our findings support that origin of inoculum of HEV-3 could influence the immune response produced in the liver. The interaction of CD68+cells with HEV-3 antigen and parenchymal NO production seems to control infection and tissue damage during convalescence, predominantly in animals infected with swine source of HEV.

Controversially, we failed to detect double CD68+iNOS+ labeled cells and HEV+iNOS+. CD68+iNOS+ labeled cells was previously described by our group in NHP experimentally infected with hepatitis A virus [27]. Results of our present study reinforce the hypothesis of macrophage/monocyte deactivation by HEV ORF3 system by inhibiting various PRRs-mediated NF-κB, thus signaling proinflammatory pathways [28][29] including IFN-γ production, which is essential for controlling HEV replication [30].

Histopathology showed a mild to moderate proliferative capacity of Kupffer cells to HEV Ag, found mainly in Kupffer cells and liver sinusoidal lining cells. Similarly, in non-viremic wild boars with naturally occurring HEV-3 infection, HEV antigen was mainly detected in Kupffer cells and liver endothelial cells, two cell populations with antigen-presenting function residing within the sinusoidal vascular space [12]. In our study, Kupffer cells, lower iNOS producer, failed to clear swine HEV-3 antigen from liver parenchyma up to the end of the study. Other authors have also described the persistence of HEV Ag in liver sinusoidal lining cells, mainly circulating sinusoidal cells, although not associated with typical inflammatory liver injury from non-viremic pigs infected experimentally [31]. These findings are relevant for public health policies since swine and NHP represent the most suitable candidate species for xenotransplant currently. [14][15].

Intrahepatically, CD4+ cells, CD4 T lymphocytes (CD3+CD4+), CD8+cells, and CD8 T lymphocytes (CD3+CD8+ cells) were detected circulating in sinusoidal space, and in focal and periportal inflammations. Both, swine- and human-HEV-3 inoculated monkeys displayed elevated CD4T/CD8T rates compared to control. Similarly, intrahepatic infiltrating T lymphocytes are present in livers biopsies from acute hepatitis E patients suggesting an additional immune-mediated mechanism of HEV-induced liver damage [5][32]. Peripherically, high IFN-γ expression in HEV-ORF2 stimulated PBMCs from patients with acute hepatitis E has been described [32]. Compartmentalization of intra-hepatic immune response could explain the weak immune reactivity of peripheral blood mononuclear cells (PBMC) during HEV infection, as in hepatitis C infection [33], where weak immune reactivity of PBMC is possibly related to the accumulation of immune events into the intrahepatic microenvironment, target of viral replication.

Moreover, the ability of HEV to replicate in extrahepatic sites may contribute to HEV persistence in liver parenchyma. Recently, HEV persistence was confirmed in bone marrow samples of cynomolgus monkeys with naturally occurring HEV infection and also in immunocompromised cynomolgus infected experimentally [34], as well as in solid organs transplant recipients [35]. However, few authors have reported HEV reactivation after immunosuppressive therapy [36].

There is still limited information available on the evaluation of intrahepatic immunological events for hepatitis E, especially during recovery. Our results also showed striking differences of immunoreactivity in the liver among infected animals regarding the source of inoculum. We suggest that intrahepatic immune response and the resolution of hepatitis E may be affected by the phylogenetic distance between the donor’s species of inoculum (swine or human) and the recipient (primates) experimentally infected. Susceptibility of related host species are known to be similar, assuming the "phylogenetic clade effect" phenomenon, which explains that hosts from a same clade shared immune response competences acquired against a specific agent [37][38]. Accordingly, we observed that human HEV-3 inoculated monkeys seem to resolve hepatitis E infection faster than those that received HEV-3 isolated from pigs. However, considering our limited number of animals inoculated with human-derived HEV-3, the influence of the source of inoculum in the response to hepatitis E requires further investigation.

In conclusion, our study confirmed the hypothesis of HEV inflammatory reactivity in the liver, even under spontaneous cure of hepatitis E infection. Long-term liver histological follow-up of experimental HEV infected cynomolgus (more than six months) should be performed to confirm the persistent outcome of the disease in immunocompetent NHP. Translationally, our results highlight the risk of HEV-3 transmission by liver transplantation, as we confirm its presence in the primate liver. Finally, we recommend a strict examination of HEV infection markers on liver implants as a protective public health measure to avoid hepatitis E spread.

## Supporting information

S1 Table HEV infection in cynomolgus monkey: Liver inflammatory score at necropsy (67 dpi). (PDF)

S2 Table Cell types staining quantification: Raw data. (XLSX)

S1 Fig Detection of HEV RNA by Nested-PCR. 1. Molecular weight marker; 2. Positive control of liver infected with HEV; 3. Bile of animal I3; 4. Gallbladder of animal I3; 5. Gallbladder of animal O1 and 6. Liver of animal O1.
(TIF)
