# Supplementary material for: Evidences of HEV genotype 3 persistence and reactivity in liver parenchyma from experimentally infected cynomolgus monkeys (Macaca fascicularis)
Source: PLoS One. 2019 Jun 18;14(6):e0218472. doi: 10.1371/journal.pone.0218472 (PMC6581283; doi:10.1371/journal.pone.0218472)
Supplement: S1 Table — (PDF) [file pone.0218472.s001.pdf]

S1 Table - HEV infection in cynomolgus monkey: liver inflammatory score at necropsy (67 dpi)

Inoculum	Animal	Score	Histophatological findings at necropsia (67 dpi)
Swine HEV-3	I3	3	Inflammatory focus +2; Ito +2
	Q11	1	Inflammatory focus +1; microsteatosis +1
	X15	2	Tumefaction +1; inflammatory focus +1; portal Inflammation +2; steatosis +1
	O1	1	Microsteatosis +1; Ito +1; portal Inflammation +1; apoptosis +1
	G3	1	Vacuolization Z3 +2; Microsteatosis +1
	F3	1	Microsteatosis +1: Ito +1; portal Inflammation +1; vacuolization Z3 +2
Human HEV-3	R7	0	Microsteatosis +2; steatosis +1; inflammatory focus +1; portal Inflammation +1
	J3	1	Microsteatosis +1; ito +1; inflammatory focus +1
Control	I2	0	Microsteatosis +1; ito +1; inflammatory focus +1
	Q12	0	Microsteatosis +1; ito +1; inflammatory focus +1
