# Supplementary figures and images for: Evidences of HEV genotype 3 persistence and reactivity in liver parenchyma from experimentally infected cynomolgus monkeys (Macaca fascicularis)
Source: PLoS One. 2019 Jun 18;14(6):e0218472. doi: 10.1371/journal.pone.0218472 (PMC6581283; doi:10.1371/journal.pone.0218472)

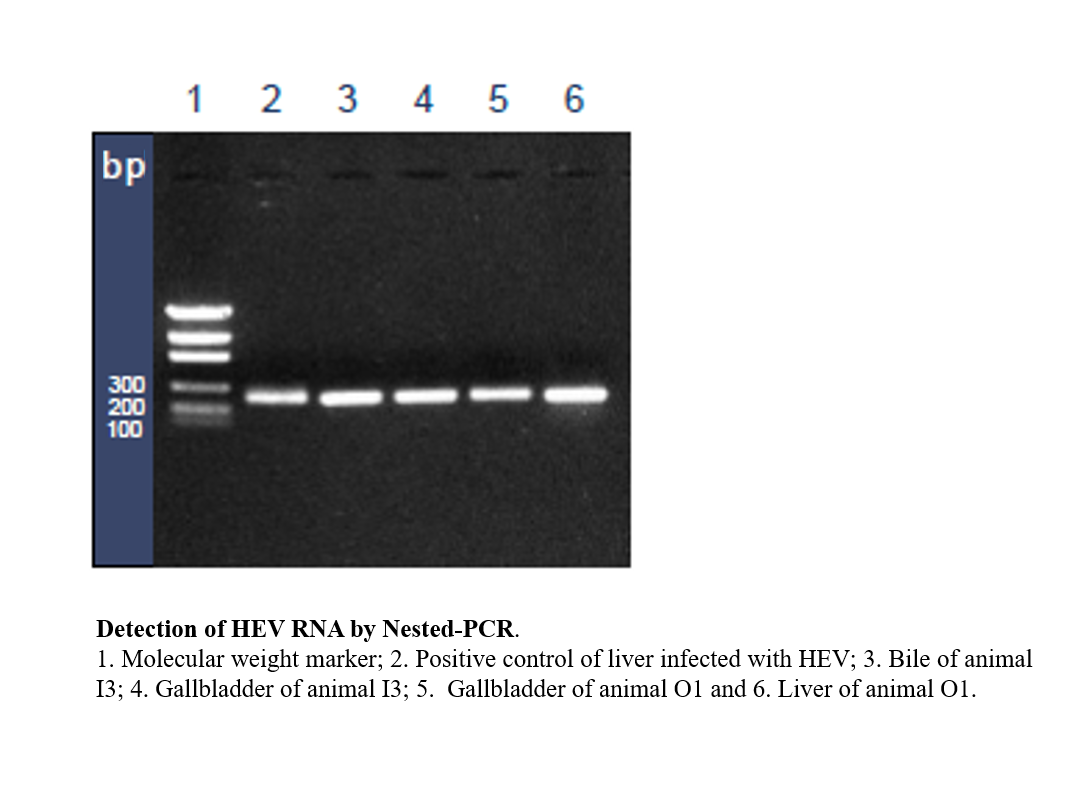

Supplement: S1 Fig — 1. Molecular weight marker; 2. Positive control of liver infected with HEV; 3. Bile of animal I3; 4. Gallbladder of animal I3; 5. Gallbladder of animal O1 and 6. Liver of animal O1. (TIF) [file pone.0218472.s003.tif]
